# Peer mentoring to support career advancement among underrepresented minority faculty in the programs to increase diversity among individuals engaged in health-related research (PRIDE)

**DOI:** 10.1017/cts.2023.535

**Published:** 2023-04-24

**Authors:** Taylor M. Coleman, Athena Starlard-Davenport, Oluwatoyosi A. Onwuemene, Lara M. Stepleman, Betty S. Pace

**Affiliations:** 1 Department of Psychiatry and Health Behavior, Medical College of Georgia at Augusta University, Augusta, GA, USA; 2 Department of Genetics, Genomics and Informatics, University of Tennessee Health Science Center, Memphis, TN, USA; 3 Division of Hematology, Department of Medicine, Duke University Medical Center, Durham, NC, USA; 4 Department of Pediatrics, Medical College of Georgia at Augusta University, Augusta, GA, USA

**Keywords:** Peer mentoring, underrepresented minority faculty, career development, mentoring competency assessment, hematology

## Abstract

Although mentoring is critical for career advancement, underrepresented minority (URM) faculty often lack access to mentoring opportunities. We sought to evaluate the impact of peer mentoring on career development success of URM early career faculty in the National Heart Lung and Blood Institute-sponsored, Programs to Increase Diversity Among Individuals Engaged in Health-Related Research-Functional and Translational Genomics of Blood Disorders (PRIDE-FTG). The outcome of peer mentoring was evaluated using the Mentoring Competency Assessment (MCA), a brief open-ended qualitative survey, and a semi-structured exit interview. Surveys were completed at baseline (Time 1), 6 months, and at the end of PRIDE-FTG participation (Time 2). The following results were obtained. Between Time 1 and Time 2, mentees’ self-assessment scores increased for the MCA (p < 0.01) with significant increases in effective communication (p < 0.001), aligning expectations (p < 0.05), assessing understanding (p < 0.01), and addressing diversity (p < 0.002). Mentees rated their peer mentors higher in the MCA with significant differences noted for promoting development (p < 0.027). These data suggest that PRIDE-FTG peer mentoring approaches successfully improved MCA competencies among URM junior faculty participants with faculty ranking peer mentors higher than themselves. Among URM faculty, peer mentoring initiatives should be investigated as a key strategy to support early career scholar development.

## Introduction

For underrepresented minority (URM) faculty pursuing academic careers, enhanced mentoring opportunities in research training are lacking [1]. URM research mentoring initiatives are being pursued both locally and nationally, including the University of San Diego’s National Center of Leadership in Academic Medicine [2,3], the multi-institutional Mid-Atlantic Center for AIDS Research Consortium Scholars Program [4], and the Federally funded National Research Mentoring Network [5]. Understanding the factors that enhance research training success for URM faculty is critical for scaling access to these opportunities at both the institutional and national levels.

A national initiative that has succeeded in providing consistent mentoring support for URM faculty is the National Heart Lung and Blood Institute (NHLBI)-sponsored Programs to Increase Diversity Among Individuals Engaged in Health-Related Research (PRIDE) [1,6,7]. Formerly the Summer Institute Program to Increase Diversity, the PRIDE program has successfully facilitated research mentoring for URM faculty through skills development, research experiences, and intense mentoring activities during focused summer institutes [7]. Of the eight summer institutes established since 2006, the PRIDE-Functional and Translational Genomics of Blood Disorders (PRIDE-FTG) program at Augusta University, has consistently sought to enhance research mentoring opportunities in basic and clinical hematology, a discipline in which the number of senior faculty available for research mentoring is limited [6].

To enhance research mentoring strategies in hematology, an important innovation of the PRIDE-FTG program is the central role of peer mentoring, which has been demonstrated to increase productivity and the efficiency and focus of research projects [8]. The PRIDE-FTG peer mentoring program improves access to mentoring, facilitates grant writing, and enhances strategies for promotion and tenure [9]. The success of this peer mentoring program in fostering community among URM faculty participants has been previously reported [9]. In this manuscript, we report the innovative strategies by which the PRIDE-FTG peer mentoring program has led to an increase in the skill of mentees and their peer mentors over the course of program participation.

## Materials and Methods

The present study was a mixed-methods evaluation study that was approved by the Institutional Review Board (IRB) of Augusta University.

### Description of the PRIDE-FTG Program

In the USA, nine unique PRIDE Programs are funded along with a central coordination core. These mentored-research programs address the difficulties experienced by URM junior investigators in establishing independent research projects and achieving higher academic ranks. The PRIDE-FTG at Augusta University, established in 2011, aims to enhance basic and clinical/translational research skills in hematology using functional and translational genomics techniques. Matriculation into the PRIDE-FTG program involves a two-stage application process including a competitive review. The program consists of two in-person Summer Institutes (SI), each lasting 10–14 days, followed by the receipt of a certificate of completion from NHLBI. Each SI trains 8–10 mentees who are assigned a primary research mentor, institutional mentor, and peer mentor as described below. Both SIs are comprised of didactic lectures, hands-on lab practical, grant-writing workshops, and an opportunity for mentees to compete for Small Research Project pilot funding to support future extramural grant applications.

### Description of the Peer Mentoring

During SI1, mentees were given a 2-hour didactic interactive lecture on peer mentoring. Immediately afterwards, they participated in a speed-mentoring event, intended to mimic speed dating, where mentees asked questions of other mentees to identify compatible peer mentors. Mentees were given sample ice-breaker questions to facilitate discussions. Prior to the conclusion of SI1, peer mentor groups were formed, ranging from 2 to 4 peers per group, dependent on cohort size and preferences. Participants could tailor the format, frequency, and goals of the peer mentoring group and were required to submit monthly narrative reports of their groups’ interactions. Peer mentoring groups continued for the 1-year duration of the PRIDE-FTG program, and some peer mentoring groups continued after the conclusion of the program. We were interested in peer mentoring self-assessment of competency over the course of the PRIDE program as well comparisons of peer mentoring competency in self-versus others. Past research has identified discrepancies in mentoring competency assessment with difference when one is rating self or others [10].

### Study Participants

The PRIDE-FTG peer mentoring program was established in 2015. Forty-four mentees from Cohorts 4 through 8 (2015–2020) were included in this study. At the time of their entry into PRIDE-FTG, mentees were 93% female, 89% Black, and 86% assistant professors.

### Evaluation Instruments

The peer mentoring aspect of the PRIDE-FTG program was evaluated using four assessments. The first two were the Mentoring Competency Assessment (MCA) [10], which were administered: (1) as a self-evaluation of mentees’ skills as a peer mentor and (2) as an evaluation of their selected peer mentor’s skills. Both MCA versions contained 26 questions, which were broken up into six major competency subscales: effective communication, aligning expectations, assessing understanding, fostering independence, addressing diversity, and promoting development (Table 1). The MCA is rated on a 7-point scale, where 1 = Not at all skilled, 4 = moderately skilled, and 7 = extremely skilled. Both the MCA evaluation of a peer mentor and the MCA self-evaluation have previously been reported in the literature to have excellent internal reliability (α = 0.95 and α = 0.91, respectively), and were found to have acceptable goodness-of-fit (CFI (comparative fit index) = 0.87, RMSEA (Root Mean Square Error of Approximation Index) = 0.080 and CFI = 0.85, RMSEA = 0.069, respectively) [10]. Our own coefficient alphas were found to be similarly excellent (Self Time 1: α = 0.96, Peer-Mentor: α = 0.99; Table 2). The third assessment was an author-derived brief open-ended qualitative question that asked for any improvement suggestions for the peer mentoring program. The final assessment was a semi-structured exit interview about participants’ overall PRIDE-FTG program experience.

**Table 1.** Mentoring Competency Assessment (MCA) competency items

MCA competencies	Skills within competencies
Effective communication	Active listening
	Providing constructive feedback
	Developing a trusting relationship
	Accommodating communication style
	Pursuing strategies to improve communication
	Coordinating with other mentors
Aligning expectations	Setting clear relationship expectations
	Aligning expectations
	Considering mentor–mentee differences
	Setting research goals
	Developing strategies to meet goals
Assessing understanding	Assessing mentee knowledge
	Estimating mentee ability
	Enhancing mentee skills
Fostering independence	Motivating mentees
	Building confidence
	Stimulating creativity
	Acknowledging mentees’ professional contributions
	Negotiating path to independence
Addressing diversity	Accounting for biases and prejudice
	Accounting for different backgrounds of mentors and mentees
Promoting development	Helping network effectively
	Setting career goals
	Helping establish a work/life balance
	Understanding impact as role model
	Helping mentees acquire resources

Note: Adapted from Fleming *et al*. [10].

**Table 2.** Comparison of Mentoring Competency Assessment (MCA) self assessment over time and of MCA self vs peer mentor assessment

Instrument	α	Possible range	N	Time 1 mean	Time 1 std dev	Time 2 mean	Time 2 std dev
*MCA mentor self-assessment*	0.96	25–175	41	5.37	0.96	5.88	0.57
Maintaining effective communication*	0.66	6–42	41	5.50	0.63	6.05	0.52
Aligning expectations*	0.88	5–35	41	5.34	1.20	5.89	1.98
Assessing understanding*	0.88	2–14	38	4.86	1.47	5.74	0.75
Fostering independence	0.89	5–35	41	5.68	1.03	5.99	0.60
Addressing diversity*	0.73	2–14	37	5.50	1.10	6.11	0.59
Promoting professional development	0.85	5–35	41	5.17	1.44	5.55	1.00

Instrument	α	Possible range	N	Self mean	Self std dev	Peer mean	Peer std dev
*MCA assessment of a peer mentor*	0.99	25–175	39	5.87	0.56	6.12	1.13
Maintaining effective communication^+^	0.96	6–42	39	6.03	0.52	6.29	0.99
Aligning expectations	0.97	5–35	38	5.86	0.66	5.95	1.20
Assessing understanding	0.97	2–14	36	5.71	0.82	5.90	1.42
Fostering independence	0.97	5–35	38	5.98	0.59	6.25	1.21
Addressing diversity	0.87	2–14	35	6.10	0.62	6.31	1.10
Promoting professional development*	0.98	5–35	39	5.58	0.98	6.01	1.34

Std dev, standard deviation.

* These scales showed a significant difference (p < .05).

+ These scales show a trend toward significance (p < .10).

### Evaluation Procedures

At the start of PRIDE-FTG SI1, mentees were consented by a member of the evaluation team. All but the last cohort included in this sample were consented in person and were given an informed consent to sign and return to PRIDE-FTG staff and a copy was given to them to keep. Due to the COVID-19 pandemic, which led to a virtual SI1 in 2020, the last cohort included in this sample was consented via Zoom (Zoom Video Communications, Inc., San Jose, CA). These participants were emailed copies of their informed consent and instructed to sign and return them as a scanned document or photo image.

### Survey Administration

After informed consent was obtained, mentees were emailed a link to the first survey on Qualtrics (Qualtrics, Provo, UT), a cloud-based surveying program. The first survey, noted as Time 1, consisted of the MCA Self-Assessment and was 5–10 minutes long. Six months into the PRIDE-FTG program, mentees were emailed a second survey that contained a qualitative question requesting suggestions for any improvements to the PRIDE-FTG peer mentoring program. The final survey administration, noted as Time 2 and given at the end of the PRIDE-FTG program participation, consisted of the MCA Self-Evaluation and the MCA Peer Mentor evaluation, lasting approximately 10–20 minutes. Also, at the conclusion of SI2, mentees completed a 30–45-minute interview with a member of the evaluation team. Three of the five cohorts included in this sample were interviewed in person. Recordings were obtained using a digital voice recorder and later transcribed by a member of the evaluation team. Both the audio recordings and the transcriptions were uploaded to Box (Box, Redwood City, CA), a Health Insurance Portability and Accountability Act-compliant cloud-based storage program. Due to the COVID-19 pandemic, the latter two cohorts completed their exit interviews virtually via Microsoft Teams (Microsoft Corporation, Redmond, WA), through which interviews were recorded and transcriptions automatically generated. Transcriptions were downloaded from Microsoft Teams and cleaned for analysis. Both the recording and transcription were uploaded to a secure cloud-based storage server.

### Analysis Plan

Quantitative survey responses were downloaded from Qualtrics and imported into IBM SPSS Statistics Software (IBM Corporation, Armonk, NY), version 28. Frequencies were used to describe demographic information of the five cohorts. Analyses for quantitative responses included paired samples t-tests for the MCA Self-Assessment at Time 1 and 2 and for comparisons between the MCA Self-Assessment at Time 2 and the MCA Assessment of a Peer Mentor (Table 2). In conjunction with quantitative analyses, qualitative responses from the 6-month survey and the exit interview were included. These qualitative findings were used as exemplars to further illustrate the constructs within the MCA in the context of peer mentoring.

## Results

### MCA Self-Assessment

**Figure 1.**
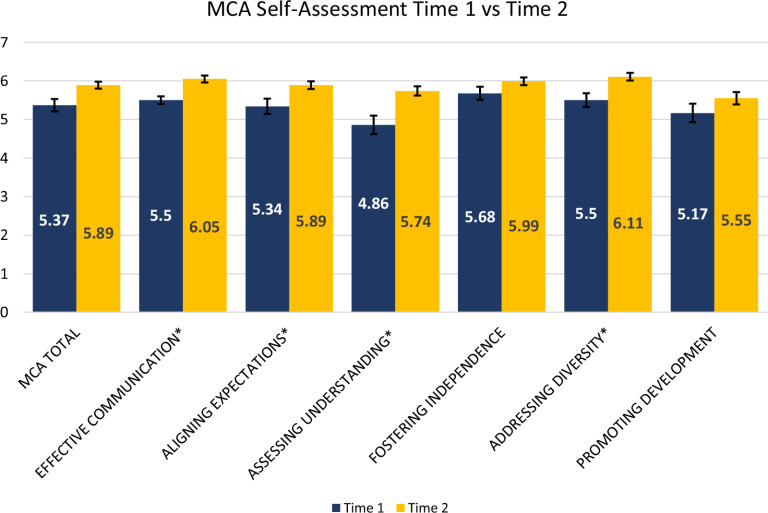
Mentoring competency assessment (MCA) differences between Time 1 and 2 self-assessment scores with denotations for statistically significant differences.

Between Time 1 and 2, participants’ self-assessment scores increased for both the total MCA and for each of the six competencies. Statistically significant increases were demonstrated for the entire MCA (p < 0.01), as well as with four of the six competencies (Table 2 and Fig. 1). Between Time 1 and 2, effective communication self-assessment scores increased significantly (p < 0.001). As an example of a component of effective communication, one participant mentioned their experience developing a trusting relationship with their peer mentees:

“*I am in a group with two other women who are called the fab three, and we continually talk or text each other just to see how each other is doing, seeing how things are going although sometimes we forget but then somebody might text and you kind of remember oh, here’s my sister in science over here, she’s my advocate so we’ve continued to [give] feedback and encourage one another so it’s really helpful.”* (Mentee 18)

Also increased between Time 1 and 2 was aligning expectations (p < 0.05). An aligning expectations characteristic is the consideration of mentor–mentee differences, which was underscored by one participant who noted:

“*Additionally, although my peer mentoring group and I converse regularly, the conversations that we ALL have via the GroupMe have been more valuable. It allows all cohort members to contribute to any point of discussion, thus adding a variety of perspectives*” (Mentee 12).

Also, significantly higher at Time 2 was assessing understanding (p < 0.001). A component of this competency, assessing mentees’ knowledge and skills, was exemplified by the following response:

“*We also read each other’s grants as well. That’s has been a great learning point for me to be able to read other people’s grants*” (Mentee 31).

Lastly, between Time 1 and 2, self-assessment scores significantly increased for addressing diversity (p < 0.01). As an example of an element of the addressing diversity subscale, one participant mentioned the benefits of working with a diverse group of peer mentees, particularly in the wake of the killings of Ahmaud Arbery, George Floyd, and Breonna Taylor:

“*Every time I got together with my peer groups, we were able to sort of let it out here, down at a whole different level, in terms of how things were affecting us. You know it was such a heavy time to be honest with you, I never ever want to talk about it again… but it was enough interactions that allowed me to actually let go and sort of start talking about that with a different group of people from what my normal environment would have exposed me to*” (Mentee 40).

### MCA Self vs. Peer Assessments

**Figure 2.**
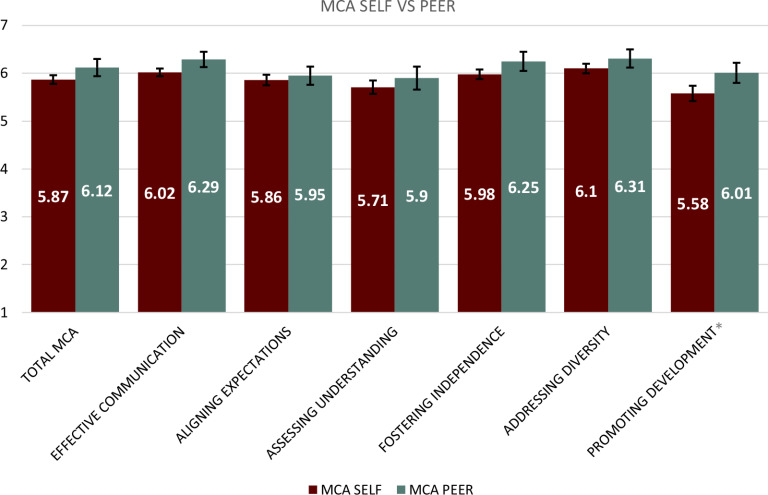
Mentoring competency assessment (MCA) differences between Time 2 self-assessment scores and peer mentor assessment scores with denotations for statistically significant differences.

When comparing the Time 2 self-assessment with the peer mentor assessment, PRIDE-FTG mentees rated their peer mentors higher in every competency and the MCA as a whole, when compared to themselves (Table 2 and Fig. 2). However, a significant difference was only seen in promoting development (p < 0.05). As an exemplar of promoting development, one mentee mentioned their peer mentors’ assistance in their career development:

“*I think I've gained confidence overall in my career advancement in my career trajectory, and… [my] peer group has also made my goal seem more attainable to me*” (Mentee 31).

## Discussion

The need for enhanced mentoring opportunities for research training of URM faculty in the biomedical sciences is well documented [11]. Mentoring is critical for career advancement, including faculty promotion and retention [12]; yet, URM faculty often receive less mentoring than their nonminority peers [13–15]. According to the Association of American Medical Colleges (AAMC), of medical school basic science full-time faculty appointed to the rank of professor, URMs make up between 3% and 4% [12]. These numbers are especially disheartening because, over the last five decades, the percentage of URM professors in medical schools basic science departments has only increased from 2% to 4% [12].

Considering the importance of mentoring for career advancement among URM faculty, we sought to evaluate the impact of peer-to-peer mentoring among five cohorts of URM early career faculty participants in the PRIDE-FTG program. Of the MCA competencies completed by program mentees between Time 1 and 2, we found that the most significant improvements, based on the mentees self-assessment, occurred with effective communication (p < 0.001), aligning expectations (p < 0.05), assessing understanding (p < 0.01), and addressing diversity (p < 0.002). However, when comparing themselves to their peer mentors, the most significant differences were noted for promoting development (p < 0.027).

The findings of the peer mentors’ report of effective communication, aligning expectations, assessing understanding, and addressing diversity as critical self-assessment competencies is not surprising. Other studies point to the importance of effective communication and diversity in developing peer mentoring relationships among junior faculty members [16–18]. Members of one PRIDE-FTG cohort published their perspective of the PRIDE-FTG experience and the impact of the peer mentoring program [9]. Similarly, other studies have shown that female peer mentors or women junior faculty who share similar interests increased their ability to accomplish career goals and academic advancement [19,20]. Studies also show that faculty with peer mentoring training, regardless of sex or gender, perceived interactions with other peers as beneficial for career advancement and success [21]. Dickson *et al.* attributed long-term (6-year) peer mentor success to the balance of similarities and differences among a group of implementation scientists in similar career phases [17]. Likewise, in a multicenter, randomized study of 150 underrepresented graduate students, postdoctoral fellows, and junior faculty, those with peer mentoring training were more likely than those without training to have discussed clinical care and career plans [22].

The development of peer mentoring relationships has also been observed to promote health care careers, increase access to mentorship, and encourage meaningful mentoring relationships between URM high school and medical students [23]. Several URM faculty in the PRIDE-FTG program observed that having a peer mentoring team with whom they could relate provided a safe environment to openly and honestly discuss their frustrations with policies and events occurring at their home institutions. As evidenced by PRIDE-FTG mentee reports of grant application review and feedback, having a peer mentoring team also afforded the mentees the opportunity to enhance their research skills and knowledge and align expectations within their institutions. These findings highlight the importance of peer mentoring in providing emotional, logistical, and professional development support for early-career scholars.

We also found that the informal use of technology such as GroupMe and cell phone text messaging proved to be invaluable resources for effective communication. As a result, several long-term supportive friendships and exchange of ideas for research collaboration have arisen. However, we also acknowledge limitations in our study. Some mentees were noncompliant with program evaluations which contributed to limited sample size for data analysis. We are continuing the peer mentoring programs for future mentees, which will increase sample size and extend the follow-up period to 2 years. Larger cohorts to validate our innovative peer mentoring strategy is critical to further evaluate the program’s efficacy. Nonetheless, our findings and those of other investigators support the critical need for expansion of innovative high-impact junior faculty peer mentoring initiatives similar to the PRIDE-FTG Program and others that achieve a greater degree of communication, support, and collaboration than traditional dyadic mentor-protégé pair relationships [21,24]. Although not the focus of this current paper, many of the URM early career faculty in the PRIDE-FTG program have attained higher academic faculty rank at their institutions, secured extramural research funding, and/or obtained tenure.

In conclusion, our innovative peer mentoring program fostered community among URM junior faculty mentees. Two major themes emerged from our data analysis. Mentees experienced an increase in peer mentoring skills over the course of the PRIDE-FTG program. Mentees tended to rate themselves lower than their peers at Time 2, which may be attributable to early career individuals’ experiences of imposter syndrome, the inner experiences of self-doubt, or overestimation of peers’ competence due to relationships. Given the challenges of URM faculty retention at academic institutions, it would be important to address mentees’ underestimation of competency and skills, which might contribute to attrition at higher faculty rank. Since peer mentoring increases productivity and sustained collaborative research relationships, it is quite plausible that efforts focused on effective communication and assessing understanding and diversity can provide a safe and supportive environment for junior faculty to discuss challenges and successes in professional development. The PRIDE-FTG program will continue the peer mentoring initiatives in support of early-career scholar development among URM populations.
